# Association of sulfotransferase SULT1A1 with breast cancer risk: a meta-analysis of case-control studies with subgroups of ethnic and menopausal statue

**DOI:** 10.1186/1756-9966-29-101

**Published:** 2010-07-21

**Authors:** Yiwei Jiang, Liheng Zhou, Tingting Yan, Zhenzhou Shen, Zhimin Shao, Jinsong Lu

**Affiliations:** 1 Department of Breast Surgery, Shanghai Cancer Hospital/Cancer Institute, Fudan University; Department of Oncology, Shanghai Medical college, Fudan University, Shanghai, 200032, China

## Abstract

**Background:**

Sulfotransferase (SULT) plays an important role in the formation of estrogen which is usually conferred as a risk factor for breast cancer. Polymorphism of the SULT1A1 may be closely associated with breast cancer. However, studies on the association between polymorphism and breast cancer have yielded inconsistent results. We performed a meta-analysis including ethnic subgroup and menopausal statue subgroup to investigate the association of SULT1A1 Arg213His polymorphism with breast cancer.

**Methods:**

PubMed, EBSCO and Web of Science databases were searched for the correlative articles up to January 2010 (10362 breast cancer patients and 14250 controls). The risk (odds ratio, OR) was used to estimate the association between SULT1A1 polymorphism and breast cancer risk. All of the data from each study use either fixed-effects or random-effects.

**Results:**

We found that SULT1A1 Arg213His had no exact effect to increase the risk of breast cancer (OR = 1.07, 95% CI: 0.97-1.17, *P *= 0.164), but it did increase the risk of breast cancer among postmenopausal women in the dominant model (OR = 1.28, 95%CI: 1.04-1.58, P = 0.019). No similar effect was found among premenopausal breast cancer women (OR = 1.06, 95%CI: 0.88-1.27, P = 0.537). There was a significant increase in breast cancer risk among Asian women (OR = 2.03, 95% CI: 1.00-4.14, P = 0.051) but not Caucasian women in recessive model. There was publication bias among postmenopausal women subgroup (*P *= 0.002), however by using the trim and fill method, if the publication bias was the only source of the funnel plot asymmetry, it needed two more studies to be symmetrical. The value of Log OR did not change too much after the adjustment and the fail-safe number of missing studies that would bring the P-value changed was 17.

**Conclusions:**

We concluded that the polymorphism of SULT1A1 Arg213His might be one of the high risk factors for breast cancer in Asian women and in postmenopausal women for all races. We should point out that the publication bias among postmenopausal women may partly account for the result, but the conclusion might not affected deeply by the publication bias.

## Background

Estrogen stimulation plays an important role in human breast cancer cell growth and development. It was reported that estrogen could affect breast cancer risk through stimulating cellular proliferation and promoting tumor progression[1]. It might be important to obtain a better understanding of enzymatic mechanism in breast cancer tissues.

Enzymatic mechanism involves in the formation of estrogen including two main pathways. One is the sulfatase pathway which involves conversion of inactive estrone sulfate into active estrone[2]. Sulfotransferase (SULT) sulfonates estrone to inactive estrone sulfate (E1-S), whereas steroid sulfatase (STS) hydrolyzes estrone sulfate to estrone. Another is the aromatase pathway which converts androstenedione into estrone and aromatase inhibitor has been successfully used in breast cancer standard treatment[3]. However, it was reported that aromatase manner was five hundred times lower than sulfatase one pointed by quantitative enzymatic evaluation [4]. Besides, early study showed that the conversion of estrogen to the inactive estrogen sulfate was very essential, as serum level of unconjugated estrone (E1) or estradiol (E2) had 10-fold lower than the level of E1-S. In addition, tissue concentration of E2 in breast cancer was 10 times higher than the level in plasma. The accumulation of E2 in breast cancer was mainly caused by the over expressed STS and the decreasing of SULT expression [5].

There are three families of SULTs. They are SULT1 family which is the major "phenol" SULT, sulfating a wide range of substrates including eight subfamilies, SULT2 family and SULT4 family. SULT1A1 gene locates in chromosome 16p11.2 - p12.1. Previous study reported that exon 7 of the SULT1A1 gene contained a G to A transition at codon 213 and showed that relevant polymorphism significantly reduced its enzymatic activity [6].

For the above reasons, genetic studies of SULT polymorphisms may improve our understanding of the mechanism of SULT and enable us to screen for individuals at high risk for different cancers. However a number of studies with conflicting outcomes have been conducted on SULT polymorphism among different cancers such as lung, ovarian, prostate and bladder [7-10]. Besides that, some authors had explored the potential association between the SULT1A1 polymorphism and breast cancer risk and it had also shown inconsistent results. Kotnis' study showed that the polymorphism of SULT1A1 Arg213His might predispose carriers to lung cancers, protect against colorectal cancers and increase the risk of breast cancer to Asian women but not the Caucasian women [11]. Recently Wang et al. meta-analyzed the relationships between SULT1A1 and breast cancer risk [12] and concluded that there was no significant relationship between SULT1A1 R213 H polymorphism and the risk of breast cancer. However both meta-analysis were not perfect and may lead to underestimate the role of SULT1A1 polymorphism in breast carcinogenesis, because they did not include some eligible studies and neglected the valuable subgroup analysis such as menopausal status. It should be pointed out that there was new finding in results of the present study which was never founded in the previous. The current meta-analysis approved to be a more precise estimation which included two more studies and a subgroup analysis according to menses status which came out statistical significance.

Here we performed an updated meta-analysis which was specialized in breast cancer, including 16 studies with a subgroup analysis based on ethnicity and menopausal status, using Arg/Arg vs His/His, Arg/Arg vs Arg/His, dominant model (Arg/His+His/His vs Arg/Arg) and recessive model (His/His vs Arg/Arg+Arg/His).

## Methods

### Identification and analysis of relevant studies

Two investigators (Yiwei Jang and Liheng Zhou) independently obtained relevant articles through searches of PubMed, EBSCO and Web of Science databases using the following words: 'sulfotransferase or SULT', 'polymorphism' and 'breast cancer'. Studies had been case-control design and based on SULT1A1 Arg213His polymorphism either alone or in combination with other genes and the language of publication was restricted to English. All of the studies required study design, publication, breast cancer cases, controls selection and genotyping methods. We excluded articles on only breast cancer patients or on healthy persons and one case-series study. In the end, 10362 breast cancer patients and 14250 controls from 16 case-control studies were selected for this meta-analysis.

### Data extraction

The following data were collected from each included studies: first authors, year of publications, study population (categorized as Asian, Caucasian, African and others), sources of controls, menopausal status and the number of different genotype in all subjects.

### Statistical analysis

The risk (odds ratio, OR) was used to estimate the association between SULT1A1 polymorphism and breast cancer risk, using Arg/Arg vs His/Arg, Arg/Arg vs His/His, dominant model (Arg/His+His/His vs Arg/Arg) and recessive model (His/His vs Arg/Arg+Arg/His). For each study, the between-study heterogeneity was assessed across by the chi-square based Q statistics and I-square test. Heterogeneity was considered at either a *P*-value of < 0.50 or I-square > 50% [13]. All of the data from each study use either fixed-effects (Mantel-Haenszel's method) or random-effects (DerSimonian and Laird's method) model according to the heterogeneity result. If there is no between-study heterogeneity, the two methods provide similar results. Funnel plots and Egger's test were used to test the possible publication bias. Sensitivity analyses were performed to estimate the influence of individual studies on the summary effect. For the possible publication bias, we used trim and fill method and fail-safe number to evaluate the influence to the result. In the ethnic population analysis, statistical analysis was performed in Asian, Caucasian, African and other populations. For menopausal status, studies were divided into postmenopausal and premenopausal status. All of the analyses were performed by Stata 10.0 software (Stata Corporation, College Station, TX, USA) and Comprehensive Meta-Analysis software program (version 2.2.034, USA, 2006), using two-sided *P *values.

## Result

### Eligible studies

Based on the search strategy, 16 studies were selected. There are 8 studies focused on the menopausal status. All of the studies were divided into four ethnic categories: Asian, Caucasian, African and others. The study details are shown in the table 1. The genotype distribution is consistent with Hardy-Weinberg equilibrium but four studies [14-30]. All of the studies were published from January 2000 to January 2010.

**Table 1:** Characteristics of studies included in the meta-analysis

			**Case**			**Control**		
				
**Author**	**Population**	**Menses**	**Arg/Arg**	**Arg/His**	**His/His**	**Arg/Arg**	**Arg/His**	**His/His**
MARIE-GENICA	Caucasian	postmenopausal	1381	1332	426	2338	2430	658
Gulyaeva	Caucasian	NM	23	40	19	63	61	56
Rebbeck	Caucasian	postmenopausal	199	226		297	259	
Rebbeck	African	postmenopausal	85	59		193	153	
Yang	Asian	premenopausal	622	116	0	614	112	0
Yang	Asian	postmenopausal	299	65	0	363	58	0
Lilla	Caucasian	NM	198	169	52	374	403	107
Le Marchand	Others	NM	801	424	114	782	484	104
Jerevall	Caucasian	postmenopausal	80	121	28	84	106	38
Han	Asian	premenopausal	92	21	3	136	23	4
Han	Asian	postmenopausal	68	20	5	219	38	6
Choi	Asian	NM	796	190	0	830	215	0
Cheng	Asian	NM	439	27	2	693	47	0
Sillanpaa	Caucasian	premenopausal	145	229	106	147	221	110
Langsenlehner	Caucasian	NM	201	250	47	224	212	63
Chacko	Asian		76	56	8	95	41	4
Chacko	Asian	premenopausa	39	27		42	24	
Chacko	Asian	postmenopausa	37	37		53	21	
Tang	Others	NM	50	42	11	134	83	13
Zheng	Others	postmenopausal	55	71	29	148	136	44
Seth	Caucasian	NM	229	176	39	110	94	23

^a^NM: not mention

### Meta-analysis database

**Figure 1:**
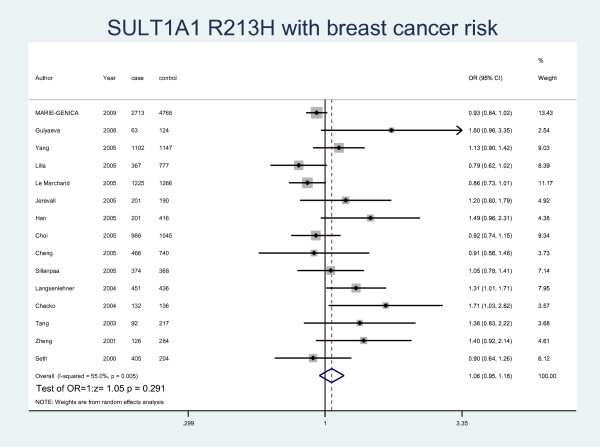
**Forest plot of meta-analysis on the association of SULT1A1 Arg213His with breast cancer risk in all population by Arg/Arg vs Arg/His model**. The size of the square box is proportional to the weight that each study contributes in the meta-analysis. The overall estimate and confidence interval are marked by a diamond. Symbols on the right of the line indicate OR > 1 and symbols on the left of the line indicate OR < 1.

**Figure 2:**
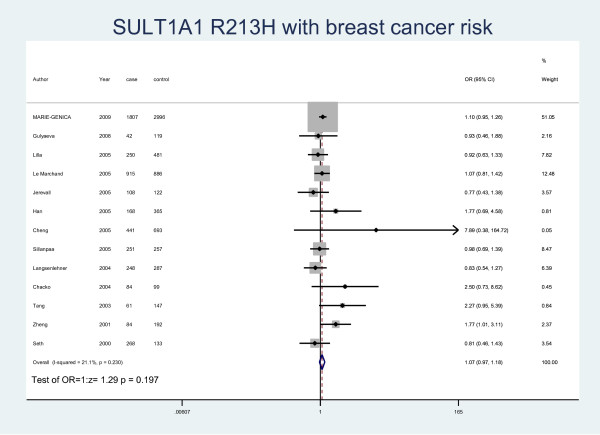
**Forest plot of meta-analysis on the association of SULT1A1 Arg213His with breast cancer risk in all population by Arg/Arg vs His/His model**. The size of the square box is proportional to the weight that each study contributes in the meta-analysis. The overall estimate and confidence interval are marked by a diamond. Symbols on the right of the line indicate OR > 1 and symbols on the left of the line indicate OR < 1.

**Figure 3:**
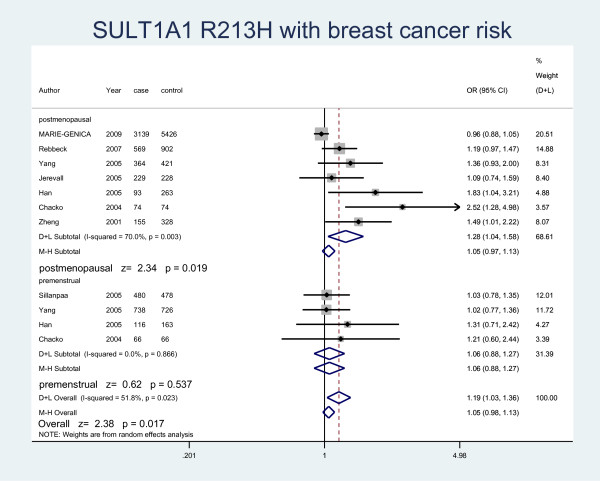
**Forest plot displaying a fixed-effects and random-effects meta-analysis on the association of SULT1A1 Arg213His with breast cancer risk by menopausal statue in the dominant model**. The size of the square box is proportional to the weight that each trial contributes in the meta-analysis. The overall estimate and confidence interval are marked by a diamond. Symbols on the right of the line indicate OR > 1 and symbols on the left of the line indicate OR < 1.

**Figure 4:**
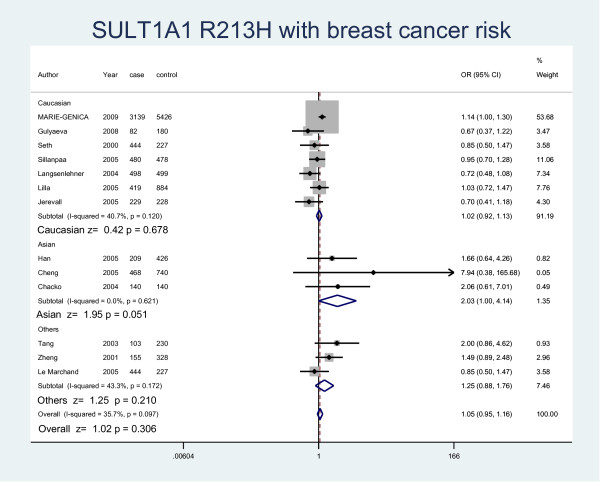
**Forest plot displaying a random-effects meta-analysis on the association of SULT1A1 Arg213His with breast cancer risk by race in the recessive model**. The size of the square box is proportional to the weight that each trial contributes in the meta-analysis. The overall estimate and confidence interval are marked by a diamond. Symbols on the right of the line indicate OR > 1 and symbols on the left of the line indicate OR < 1.

The details of the study characteristics and the ORs we calculated were listed in Table 2. In the dominant model (Arg/His+His/His vs Arg/Arg), there was between-study heterogeneity in the odds ratios (ORs) of the studies (Heterogeneity chi-squared = 30.09 (d.f. = 15), I-squared = 50.2%, *P *= 0.012), so we used the random-effect model to analyze the data and found that there was no relationship between Arg/His+His/His genotype and the risk of breast cancer (OR = 1.07, 95% CI: 0.97-1.17, *P *= 0.164). In the recessive model (His/His vs Arg/Arg+ Arg/His), there was no between-study heterogeneity in the odds ratios (ORs) of the studies (Heterogeneity chi-squared = 18.25 (d.f. = 12) I-squared = 34.3%, *P *= 0.108). Through the fixed-effect model we found that it was no relationship with breast cancer risk (OR = 1.07, 95% CI: 0.97-1.17, P = 0.169). We used random-effect model (Heterogeneity chi-squared = 31.11 (d.f. = 14) I-squared = 55.0%, *P *= 0.005) to analyze Arg/Arg vs Arg/His (OR = 1.06, 95%CI: 0.95-1.18, P = 0.291) (Fig. 1) and fixed-effect model (Heterogeneity chi-squared = 15.21 (d.f. = 12) I-squared = 21.1%, *P *= 0.230) to analyze Arg/Arg vs His/His (OR = 1.07, 95%CI: 0.97-1.18, P = 0.197) (Fig. 2), there was no relationship between SULT1A1 and breast cancer risk either. Meanwhile, we analyzed the subgroups of the studies and found that genotype Arg213His increased the risk of breast cancer among postmenopausal women (OR = 1.28, 95% CI: 1.04-1.58, P = 0.019) but not in the premenopausal women (OR = 1.06, 95% CI: 0.88-1.27, P = 0.537) by both M-H method and D-L method. Because of the different heterogeneity results for postmenopausal women (Heterogeneity chi-squared = 20.01 (d.f. = 6) I-squared = 70%, *P *= 0.003) and premenopausal women (Heterogeneity chi-squared = 0.73 (d.f. = 3) I-squared = 0.0%, *P *= 0.866), we used both M-H method and D-L method. For all the studies included in the menses subgroup (Heterogeneity chi-squared = 20.74 (d.f. = 10) I-squared = 51.8%, *P *= 0.023), there was also statistical significance (OR = 1.19, 95% CI: 1.03-1.36, P = 0.017) (Fig. 3). As for the ethnic subgroups, we used fixed-effects to analyze the studies. We found that racial difference influenced the relationship between the polymorphism and the breast cancer risk, especially in Asian women (M-H method, Heterogeneity chi-squared = 0.95 (d.f. = 2) I-squared = 0.0%, *P *= 0.621, OR = 2.03, 95% CI: 1.00-4.14, P = 0.051) but not Caucasian women (M-H method, Heterogeneity chi-squared = 10.12 (d.f. = 6) I-squared = 40.7%, *P *= 0.120, OR = 1.02, 95% CI: 0.92-1.13, P = 0.678) (Fig. 4).

**Table 2:** ORs of studies included in the meta-analysis

				**OR(95%CI)**	**OR(95%CI**	**OR(95%CI)**	**OR(95%CI)**
**Author**	**Population**	**Menses**	**Year**	**Arg/His+His/His vs Arg/Arg**	**His/His vs Arg/Arg+ Arg/His**	**Arg/Arg vs Arg/His**	**Arg/Arg vs His/His**
MARIE-GENICA	Caucasian	postmenopausal	2009	0.96(0.88-1.05)	1.14 (1.00-1.30)	0.93 (0.84-1.02)	1.10 (0.95-1.26)
Gulyaeva	Caucasian	NM	2008	1.38(0.78-2.44)	0.67 (0.37-1.22)	1.80 (0.96-3.35)	0.93 (0.46-1.88)
Rebbeck	Caucasian	postmenopausal	2007	1.19(0.97-1.47)	Excluded	Excluded	Excluded
Rebbeck	African	postmenopausal	2007				
Yang	Asian	premenopausal	2005	1.13(0.90-1.42)	Excluded	1.13 (0.90-1.42)	Excluded
Yang	Asian	postmenopausal	2005				
Lilla	Caucasian	NM	2005	0.82(0.65-1.03)	1.03 (0.72-1.47)	0.79 (0.62-1.02)	0.92 (0.63-1.33)
Le Marchand	Others	NM	2005	0.89(0.77-1.04)	1.13 (0.86-1.49)	0.86 (0.73-1.01)	1.07 (0.81-1.42)
Jerevall	Caucasian	postmenopausal	2005	1.09(0.74-1.59)	0.70 (0.41-1.18)	1.20 (0.80-1.79)	0.77 (0.44-1.38)
Han	Asian	premenopausal	2005	1.53(1.02-2.31)	1.66 (0.64-4.26)	1.49 (0.96-2.31)	1.76 (0.69-4.58)
Han	Asian	postmenopausal	2005				
Choi	Asian	NM	2005	0.92(0.74-1.15)	Excluded	0.92 (0.74-1.15)	Excluded
Cheng	Asian	NM	2005	0.97(0.60-1.57)	7.93(0.38-165.68)	0.91 (0.58-1.48)	7.89 (0.38-164.72)
Sillanpaa	Caucasian	premenopausal	2005	1.03(0.78-1.35)	0.95 (0.70-1.28)	1.05 (0.78-1.41)	0.98 (0.69-1.39)
Langsenlehner	Caucasian	NM	2004	1.20(0.94-1.55)	0.72 (0.48-1.08)	1.31 (1.01-1.71)	0.83 (0.55-1.27)
Chacko	Asian		2004	1.78(1.09-2.89)	2.06 (0.61-7.01)	1.71 (1.03-2.82)	2.50 (0.73-8.62)
Chacko	Asian	premenopausal	2004				
Chacko	Asian	postmenopausal	2004				
Tang	Others	NM	2003	1.48(0.93-2.36)	2.00 (0.86-4.62)	1.36 (0.83-2.22)	2.27 (0.95-5.39)
Zheng	Others	postmenopausal	2001	1.49(1.01-2.22)	1.49 (0.89-2.48)	1.41 (0.92-2.14)	1.77 (1.01-3.11)
Seth	Caucasian	NM	2000	0.88(0.64-1.22)	0.85 (0.50-1.47)	0.90 (0.64-1.26)	0.82 (0.46-1.43)

^a^NM: not mention

### Publication bias and Sensitivity analyses

**Figure 5:**
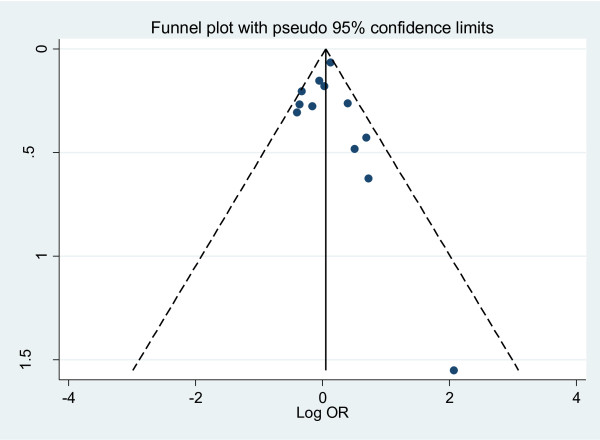
**Funnel plots for publication bias for population subgroup in recessive model**. Funnel plot of the log odds-ratio, against its standard error for publication bias in SULT1A1 Arg213His.

**Figure 6:**
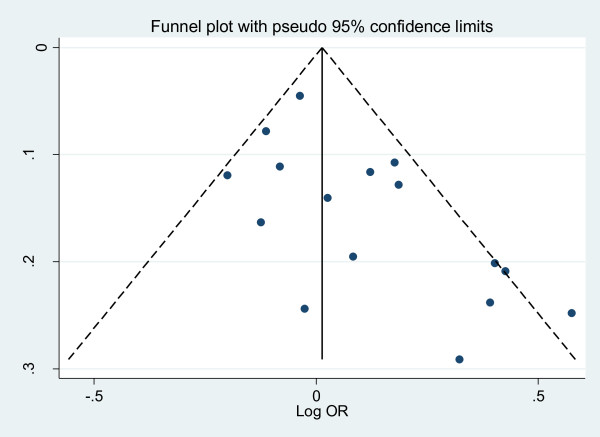
**Funnel plots for publication bias for all population in dominant model**. Funnel plot of the log odds-ratio, against its standard error for publication bias in SULT1A1 Arg213His.

**Figure 7:**
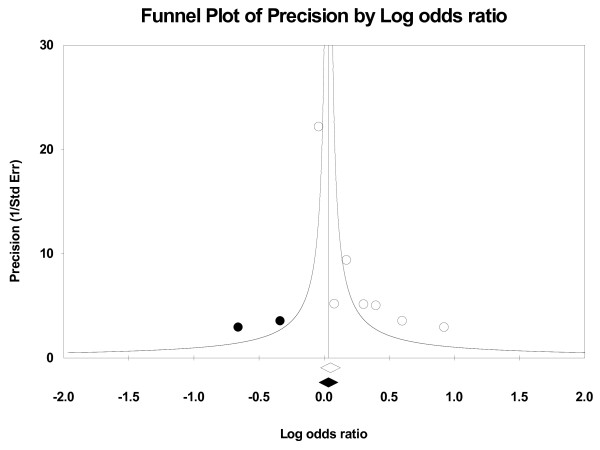
**Funnel plot of Precision by Log odds ratio**. The filled circles are missed studies due to publication bias. The bottom diamonds show summary effect estimates before (open) and after (filled) publication bias adjustment.

**Figure 8:**
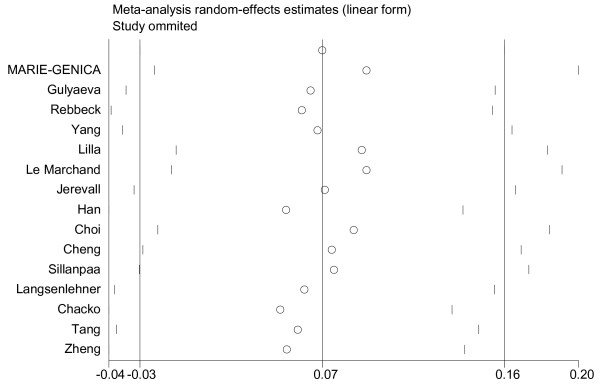
**Sensitivity analyses for the influence of individual studies on the summary effect**. Sensitivity analyses for the influence of individual studies on the summary OR. The vertical axis indicates the overall OR and the two vertical axes indicate its 95% CI. Every hollow round indicates the pooled OR when the left study is omitted in this meta-analysis. The two ends of every broken line represent the respective 95% CI.

We performed the funnel plots and Egger's test to assess the publication bias. As a result there was no publication bias in recessive model (t = 0.16, P = 0.875), Arg/Arg vs His/His model (t = 1.09, P = 0.299), subgroup for population (t = 0.02, P = 0.985) (Fig. 5). But there was publication bias for all population in dominant model (t = 2.82, P = 0.014) (Fig. 6) and Arg/Arg vs Arg/His model (t = 3.21, P = 0.007). This might be a limitation for our analysis because studies with null findings, especially those with small sample size, are less likely to be published. Also there was a publication bias (for postmenopausal women: t = 5.96, P = 0.002) as the result suggested. By using the trim and fill method, we showed that, if the publication bias was the only source of the funnel plot asymmetry, it needed two more studies to be symmetrical. The value of Log OR did not change too much after the adjustment (Fig. 7). Beside that, the fail-safe number of missing studies that would bring the P-value changed was 17. The influence of individual studies on the summary effect estimate was performed by sensitivity analyses on the overall OR (Fig. 8). No individual study affected the overall OR, since omission of any single study made no materially huge difference.

## Discussion

Prolonged exposure to high level of estrogen still has been appreciated as a risk factor for breast carcinogenesis. From previous study we knew that SULT1A1 was an important enzyme in xenobiotic metabolism because it had broad substrate specificity with a high affinity for many compounds [31,32], furthermore SULT immunoreactivity was associated with tumor size (*P *= 0.0030) or lymph node status (*P *= 0.0027) [4]. This meta-analysis with 16 studies demonstrates no significant association of SULT1A1 polymorphism with breast cancer risk in the overall study populations which is similar with the previous result [12]. One reason may be that the effect of a single nucleotide polymorphism might have a limited impact on breast cancer risk. The result indicated that multiple SNP-based approaches rather than a single nucleotide polymorphism-based strategy may provide more exact information on relationship between SULT1A1 and breast cancer. Future research should be directed to evaluate the effect of other polymorphisms. Another reason may be that SULT1A1 polymorphism has relation to breast cancer in part of the women and the whole population analysis may weaken this relationship. Therefore subgroup analysis should be done to find whether it is one of the breast cancer risk factors.

From the ethnic subgroup, we found that there was significant result among the different race. SULT1A1 R213 H increased the risk of breast cancer among Asian women but not Caucasian women in recessive model (His/His vs Arg/Arg+Arg/His) which was consistent with the previous studies. Carlsten had reported the similar phenomenon for GSTM1 polymorphism which conferred a significantly increased risk of lung cancer to East Asians but not to Caucasians[33]. The frequency of SULT1A1 allele was different among the ethnic groups. From the previous study we knew that the maximum value of the His allele frequency is 0.18 in the Asian, which was much lower than the minimum value 0.23 in the Caucasian [12]. The potential explanation is that the allele frequencies in Asian population are very low and are fairly different from those observed in Caucasian and Africans [31]. It also should be pointed out that only three studies included in this analysis. More studies needed to confirm the result.

In the subgroup analysis of different menopausal statue, we surprisingly found that SULT1A1 polymorphism increased the risk of breast cancer among postmenopausal women but not among premenopausal women. In the Yang's research, a possible association between SULT1A1 and breast cancer risk was also suggested for postmenopausal women [17]. However, two thirds of breast cancers occur during the postmenopausal period when the ovaries have ceased to be functional [32]. It was also reported that higher serum concentrations of estrogens were associated with increased breast cancer risk in postmenopausal women [34]. Early studies indicated that several factors could be implicated in this process, including higher steroids which were gained from plasma and the potent E2 which was formed by the breast cancer tissue itself [5]. However, the serum hormone levels change with the menstrual cycle and the cycle length varies individually, so it is difficult to address the association of hormone levels and breast cancer risk among premenopausal women [35]. Multiple factors may act on the breast cancer risk among premenopausal women, but our analysis supported that the polymorphism of SULT1A1 may have significant effects on the relationship between breast cancer risk and SULT1A1 Arg213His polymorphism among the postmenopausal women. Due to the publication bias we found, the result may remain uncertain. By the trim and fill method and the fail-safe number, we can find that the publication bias may have a small effect on the result. So the publication bias may partly account for the result.

There were some limitations of this meta-analysis. First, the unavailable genotype data from some articles was the main limitation. We did everything possible to obtain the full data on the subjects, and about 75 percent of subjects involved in various ethnic populations. Lack of original data of each study may prevent more detailed analyses such as joint effects of SNP-SNP which we hope will be demonstrated by the following studies. Next, some controls were selected from benign breast disease which have potential risks of developing breast cancer might lead to misclassification. These limitations may also explain the publication bias in postmenopausal women.

## Conclusion

In a conclusion, SULT1A1 Arg213His may be associated with breast cancer risk in Asian women and postmenopausal women among all races, although there are no exact effects to increase the risk of breast cancer in premenopausal women. Due to the publication bias we found, it encourages more studies to pay attention on the menopausal statue in further researches.

## Competing interests

The authors declare that they have no competing interests.

## Authors' contributions

JSL is responsible for editorial correspondence and has contributed to the conception and design of the study, the analysis and interpretation of data, the revision of the article as well as final approval of the version to be submitted. YWJ and LHZ participated in the design of the study, performed the statistical analysis, searched and selected the trials, drafted and revised the article. TTY participated in the design of the study and helped to revise the article. ZZS conceived of the study, and participated in its design and coordination. ZMS conceived of the study, and participated in its design and coordination. All authors read and approved the final version of the manuscript.
